# CD103 Expression on Regulatory and Follicular T Cells in Lymph Nodes, Bronchoalveolar Lavage Fluid and Peripheral Blood of Sarcoidosis Patients

**DOI:** 10.3390/life12050762

**Published:** 2022-05-20

**Authors:** Miriana d’Alessandro, Sara Gangi, Dalila Cavallaro, Laura Bergantini, Fabrizio Mezzasalma, Stefano Cattelan, Stefano Baglioni, Marta Abbritti, Paolo Cameli, Elena Bargagli

**Affiliations:** 1 Respiratory Diseases Unit, Department of Medical and Surgical Sciences & Neurosciences, University of Siena, 53100 Siena, Italy; dalila.cavallaro@student.unisi.it (D.C.); bergantini@student.unisi.it (L.B.); stefano.cattelan@student.unisi.it (S.C.); paolo.cameli@unisi.it (P.C.); bargagli2@unisi.it (E.B.); 2 Diagnostic and Interventional Bronchoscopy Unit, Cardio-Thoracic and Vascular Department, University Hospital of Siena (Azienda Ospedaliera Universitaria Senese, AOUS), 53100 Siena, Italy; fabrizio.mezzasalma@ao-siena.toscana.it; 3 Pneumology Department, Perugia Hospital, 06129 Perugia, Italy; stefano.baglioni@ospedale.perugia.it (S.B.); marta.abbritti@ospedale.perugia.it (M.A.)

**Keywords:** sarcoidosis, integrins, CD103, endobronchial ultrasound-guided transbronchial needle aspiration, bronchoalveolar lavage

## Abstract

(1) Background: Sarcoidosis is a chronic multisystem disorder of unknown aetiology, driven by a T-cell mechanism allowing T-cell attachment and transmigration through the endothelium, and endorsed by the expression of an integrin alpha-E beta-7 (CD103). This study aimed to analyse the different distribution and compartmentalisation of CD103 expression on T cell subsets in BAL, peripheral blood mononuclear cells (PBMC) and lymph nodes (LLN) from sarcoidosis patients. (2) Patients: We consecutively and prospectively enrolled 14 sarcoidosis patients. We collected PBMC, LLN and BAL at the same time from all patients. Through flow cytometric analysis, we analysed the expression of CD103 on regulatory and follicular T cell subsets. (3) Results: All patients were in radiological Scadding stage II. The multivariate analysis found that the variables which most influenced the peripheral blood compartment were high CD8^+^ and low ThReg, CD8^+^CD103^+^ and Tfh cell percentages. A principal component analysis plot performed to distinguish LLN, BAL and PBMC showed that they separated on the basis of CD4^+^, CD4^+^CD103^+^, CD8^+^, CD8^+^CD103^+^, TcEffector, TcNaive, ThNaive, ThEffector, Threg, ThregCD103^+^, Tfh, TcfCXC5^+^ and CD4^+^CD103^+^/CD4^+^ with 65.96% of the total variance. (4) Conclusions: Our study is the first to report a link between the imbalance in circulating, alveolar and lymph node CD8^+^ and CD8^+^CD103^+^ T cells, ThReg, Tfh and ThNaive and the CD103^+^CD4^+^/CD4^+^ T cell ratio in the development of sarcoidosis. These findings shine a spotlight on the pathogenesis of sarcoidosis and may offer new predictors for diagnosis. Our study provides additional understanding for a personalised, and hopefully more effective treatment of sarcoidosis.

## 1. Introduction

Sarcoidosis is a chronic multisystem disorder of unknown aetiology, mainly affecting the lungs, and characterised by the presence of non-caseating granulomas [1,2]. Bronchoalveolar lavage (BAL) examination, a minimally invasive tool, contributes to the diagnosis of sarcoidosis on the basis of an elevated CD4^+^/CD8^+^ T cell ratio [3,4,5,6]. Sarcoidosis is driven by a T-cell mechanism, in particular the accumulation of activated CD4^+^ T-cells in the lungs, allowing T-cell attachment and transmigration through the endothelium, endorsed by the expression of integrins [7]. Integrins are a large family of transmembrane proteins and the main receptors for extracellular matrix components [8]. Most studies on CD4^+^ and CD8^+^ T cell subpopulations have identified an integrin alpha-E beta-7 (CD103), an adhesion molecule with αE and β7 subunits [9,10]. This molecule can promote T-cell migration into the epithelium and is involved in retaining lymphocytes in the mucosa [11]. The literature on sarcoidosis reports CD103 to be stage-dependent, and mainly related to stages II and III [12]. CD103 is certainly expressed on 95% of intraepithelial CD4^+^ lymphocytes in the mucosa, but on less than 2% of circulating peripheral blood lymphocytes [6]. Sarcoidosis patients show lower CD103 expression on BAL CD4^+^ cells than patients with other granulomatous ILDs, suggesting that these cells are of peripheral origin [6,13,14].

Some data are available on CD103 expression on CD8^+^ cells regulated by TGF-beta in healthy donor cell cultures [15,16]. Such expression is not demonstrated in sarcoidosis patients. Moreover, no studies have described CD103 expression in the lymph node compartment or on T-follicular and regulatory T cells from sarcoidosis patients.

An aim of this study was to analyse the different distribution and compartmentalisation of CD103 expression on T cell subsets in BAL fluid, peripheral blood and lymph node tissue from sarcoidosis patients. Correlations between clinical and immunological features were also tested. 

## 2. Materials and Methods

### 2.1. Study Population

For this study, patients were recruited from the sarcoidosis regional referral centre at Siena University Hospital and from the respiratory unit of Perugia Hospital, Italy. Newly diagnosed sarcoidosis patients underwent simultaneous bronchoscopy with BAL, lung-draining lymph node (LLN) biopsy and peripheral blood sampling. Diagnosis according to international criteria [1] was based on clinical signs, chest radiography findings and non-caseating granulomas in lymph nodes and/or endobronchial biopsy specimens. All patients with calcified lymph nodes and those whose samples were not obtained simultaneously were excluded. Prior to bronchoscopy and biopsy (by EBUS-TBNA), high resolution computed tomography (HRCT) of the chest showed enlarged lymph nodes in all patients. 

All patients gave their written informed consent to participate in the study. Healthy donors were not enrolled for ethical reasons. The study was approved by the regional ethical review board of Siena, Italy (C.E.A.V.S.E. Markerlung 17431) and complied with the declaration of Helsinki.

### 2.2. Gating Strategy

Multicolour flow cytometric analysis was performed. The following mAb were used to detect the different subsets of T and B cells: CD3 APC-Cy7 (OKT3 Biolegend), CD8 BV421 (SK1 Biolegend), CXCR5 PerCPCy5 (J252D4 Biolegend), CD103 PE (mouse IgG1k), CD25 PE-Cy7 (2A3 BD), CD4 FITC (SK3 BD), CD127 APC (hIL-7R-M21 BD) and CD19 PE-Cy7 (SJ25C1). 

Two tubes were assessed for T and B cell subsets. The first tube was for lymphocytes discriminated on the basis of forward (FSC) versus side (SSC) scatters. Then, a dot plot was performed to identify CD3-expressing cells, and a secondary dot plot to distinguish CD4- from CD8-expressing cells. CD103-expressing populations of CD4-positive (CD4^+^CD103^+^) and CD8-positive cells (CD8^+^CD103^+^) were identified. Using CD25 and CD127 markers, a dot plot was assessed on CD4-positive cells to discriminate three different subtypes of T cells: T helper regulatory (Th-reg, CD4^+^CD25^+^CD127^−^), T helper effector (CD4^+^CD25^+^CD127^+^) and T helper naive (CD4^+^CD25-CD127^+^). Gating strategy also included another dot plot which identified a Th-reg population expressing CD103. Three different subsets of CD8-positive cells were distinguished according to CD25 and CD127 markers: T cytotoxic regulatory (Tc-reg, CD8^+^CD25^+^CD127^−^), T cytotoxic effector (CD8^+^CD25^+^CD127^+^) and T cytotoxic naive (CD8^+^CD25-CD127^+^). Another dot plot identified a Tc-reg population expressing CD103.

To distinguish T follicular cells, two dot plots were assessed according to expression of CXCR5: T follicular helper (Tfh, CD4^+^CXCR5^+^) and T follicular cytotoxic (Tfc, CD8^+^CXCR5^+^). CD103 expression was evaluated on Tfh (CD4^+^CXCR5^+^CD103^+^) and Tfc cells (CD8^+^CXCR5^+^CD103^+^).

The second tube was used to evaluate B cells according to CD19 expression. 

**Figure 1:**
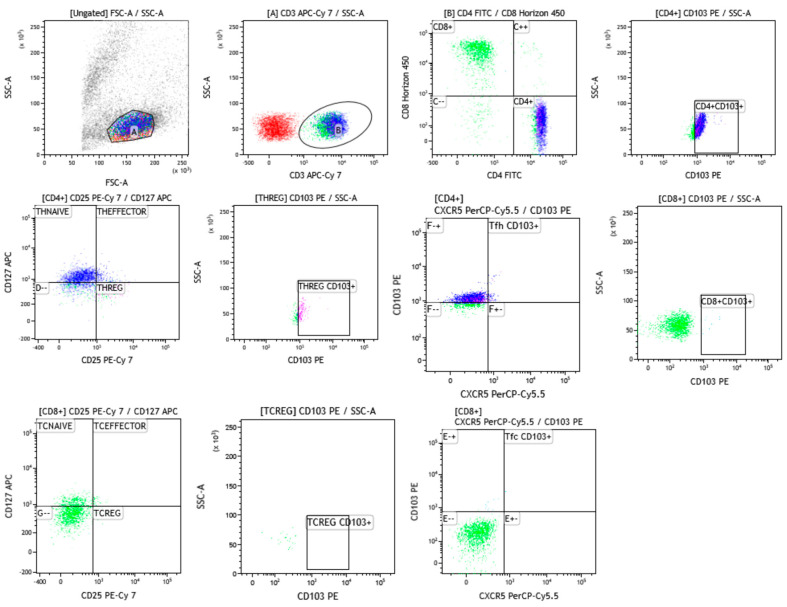
An example of gate strategy from sarcoidosis patient to identify T cell subsets and their expression of CD103. The blue population was referred to CD4-positive cells while green one to CD8-positive cells.

The gating strategy performed through Kaluza Software 2.1 (Beckman Coulter, Brea, CA, USA) was reported in Figure 1. 

### 2.3. Statistical Analysis

All values were expressed as median and interquartile range (IQR) and mean ± standard deviation, when appropriate. The normal distribution of values was determined with the Shapiro–Wilk test. Non-parametric one-way ANOVA (Kruskal–Wallis test) and Dunn test were performed for multiple comparisons. For categorical variables, Fisher’s exact or Chi-squared tests were used to compare proportions between groups. Spearman’s test was used to find correlations between clinical and immunological parameters.

Multivariate analysis adjusted for sex, age and smoking was performed on cell subsets (CD4, CD4^+^CD103^+^, CD8, CD8^+^CD103^+^, TcEffector, TcNaive, ThNaive, ThEffector, Threg, ThregCD103^+^, Tfh, TcfCXC5^+^, CD4^+^CD103^+^/CD4^+^) that differed in a statistically significant manner between BAL fluid, PBMC and LLN samples.

Supervised Principal Component Analysis (PCA) was used in an explorative approach to identify trends in immunological features by 2D representation of the multi-dimensional data set.

To understand or predict the effect of the statistically significant cell subsets in the three anatomical compartments, an ordinal logistic regression was performed, and a classification table was drawn up for the training sample (confusion matrix) and confusion plot. To investigate the best thresholds for classifying the peripheral, alveolar and lymph node compartments, three groups were formed. A classification and regression decision tree was constructed to determine the best clustering variables according to the Gini criterion [17]. We created a series of test/training partitions to evaluate the accuracy of potential binary classifiers by means of a confusion matrix. 

A *p*-value less than 0.05 was considered statistically significant. Statistical analysis was performed by GraphPad Prism 9.3 and XLSTAT 2021 software.

## 3. Results

### 3.1. Study Population

We enrolled 14 patients with sarcoidosis (median age of 55 (53–59) years) consecutively and prospectively. We collected PBMC, LLN and BAL samples at the same time from all patients. Immunological findings are reported in Table 1. Seven patients were females, and there was a prevalence of never-smokers (57%). All patients were in Scadding radiological stage II indicating lymph node involvement in addition to granulomas in the lungs. None were on treatment at the time of bronchoscopy and sampling. Lymphocytosis (median, IQR 20 (9–38)) and a high CD4/CD8 ratio (median, IQR 4.79 (2.36–6.75) were recorded in BAL samples from all patients.

### 3.2. CD4, CD8, CD19 and CD103 Expression in LLN, BAL and PBMC Samples

CD4^+^ cell percentages were higher in LLN than PBMC samples (*p* = 0.0259), while CD8^+^ cell percentages were lower in LLN than PBMC samples (*p* = 0.0023). The CD4^+^/CD8^+^ ratio was higher in LLN than PBMC samples (*p* = 0.0084). Double-positive cells expressing CD4 and CD103 were higher in PBMC than LLN (0.0129) and BAL samples (*p* = 0.0180). As well, the CD4^+^CD103^+^/CD4^+^ ratio was higher in PBMC than LLN (0.0008) and BAL samples (*p* = 0.0025). Double-positive cells expressing CD8 and CD103 were higher in BAL than PBMC (*p* = 0.0382) and LLN samples (*p* = 0.0446). There were inverse correlations between age and CD4-positive cells (r = −0.447, *p* = 0.0249) and CD4^+^CD103^+^ (r = −0.0473, *p* = 0.0196). CD19^+^ cell percentages were lower in BAL than LLN (*p* = 0.0015) and PBMC (*p* = 0.0129) samples. However, B cells were higher in LLN than PBMC samples (*p* = 0.0354).

### 3.3. T Regulatory Cells and CD103 Expression in LLN, BAL and PBMC Samples

Th effectors were lower in BAL than in LLN (*p* = 0.0150) and PBMC samples (*p* = 0.0095); the same was true for Th naïve (*p* = 0.0266 and *p* = 0.0179, respectively). Treg were lower in PBMC than in BAL (*p* = 0.0484) and LLN samples (*p* = 0.0490), whereas Treg expressing CD103 were higher in PBMC than in BAL (*p* = 0.0062) and LLN samples (*p* = 0.0279). 

Tc naïve were higher in LLN than in BAL samples (*p* = 0.0410); Tc effectors were higher in LLN than in PBMC samples (*p* = 0.0096) and were not detected in BAL samples. There were no significant differences between Tcreg cells in LLN, BAL and PBMC samples, whereas Tcreg cells expressing CD103 were detected only in BAL samples. 

### 3.4. T Follicular Cells and CD103 Expression in LLN, BAL and PBMC Samples

Tfh cells were higher in LLN than in BAL (*p* = 0.0185) and PBMC samples (*p* = 0.0036), and higher in BAL fluid than in PBMC (*p* = 0.0103). There were no significant differences in Tfh cells expressing CD103 in LLN, BAL or PBMC samples. Tfc cells were higher in LLN than in PBMC samples (*p* = 0.0305) but were not detected in BAL samples. However, Tfc cells expressing CD103 were higher in BAL than in LLN (*p* = 0.0471) and PBMC samples (*p* = 0.0399). 

### 3.5. Multivariate Analysis

**Figure 2:**
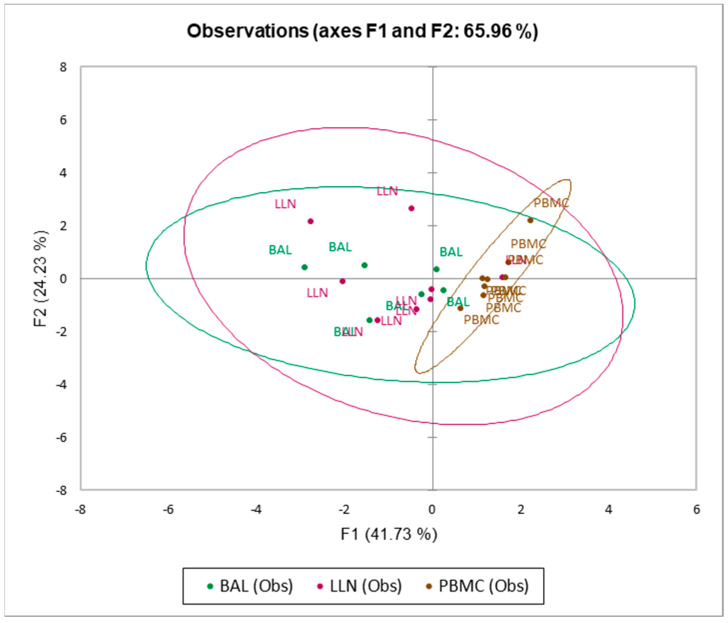
Principal component analysis (PCA) to distinguish the three groups lymph nodes (LLN), bronchoalveolar lavage (BAL) and peripheral blood mononuclear cells (PBMC). The first and second components explained 65.96% of the total variance.

The PCA plot (Figure 2) performed to distinguish the three groups LLN, BAL and PBMC showed that they separated on the basis of CD4^+^, CD4^+^CD103^+^, CD8^+^, CD8^+^CD103^+^, TcEffector, TcNaive, ThNaive, ThEffector, Threg, ThregCD103^+^, Tfh, TcfCXC5^+^ and CD4^+^CD103^+^/CD4^+^. The first and second components explained 41.73% and 24.23% of the total variance.

**Figure 3:**
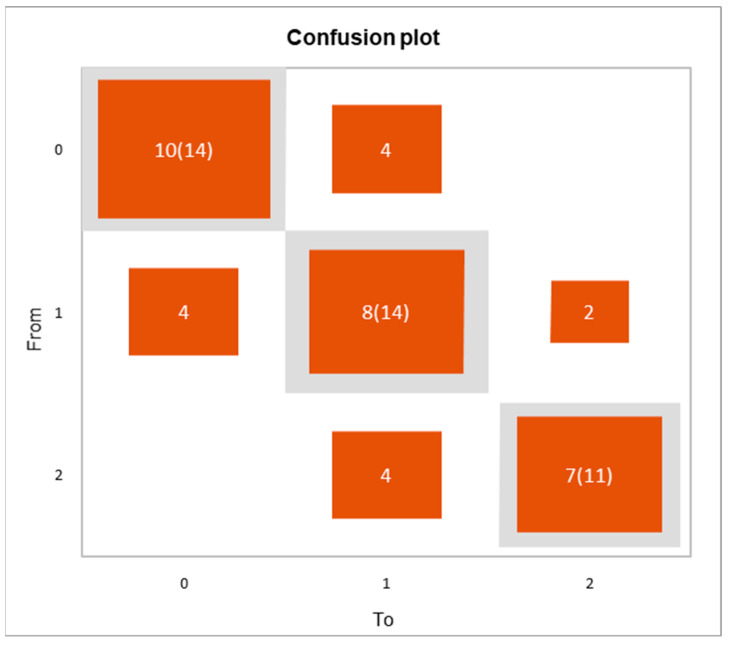
Confusion plot reporting the variable 0 (LLN samples) was well classified at 71.4%, while variable 1 (BAL samples) and variable 2 (PBMC samples) were well classified at 57.1% and 63.6%. The grey squares on the diagonal represent the observed numbers for each modality. The orange squares represent the predicted numbers for each modality. The Goodness of Classification Index showed that 26.9% of the observations were well classified.

The goodness-of-fit statistics showed a Chi^2^ associated with a Log ratio of 0.003, indicating that the variables bring a significant amount of information. From the probability associated with the Chi-square tests, the Type II analysis showed that the variables that most influenced the three differential compartments were CD8, ThReg, CD8^+^CD103^+^ and Tfh (*p* = 0.049, *p* = 0.035, *p* = 0.044 and *p* = 0.020, respectively). The LLN samples were well classified at 71.4% while BAL and PBMC samples were well classified at 57.1% and 63.6%, respectively (Figure 3). The Goodness of Classification Index showed that 26.9% of the observations were well classified, which means that the predictive quality of this classification model is good.

**Figure 4:**
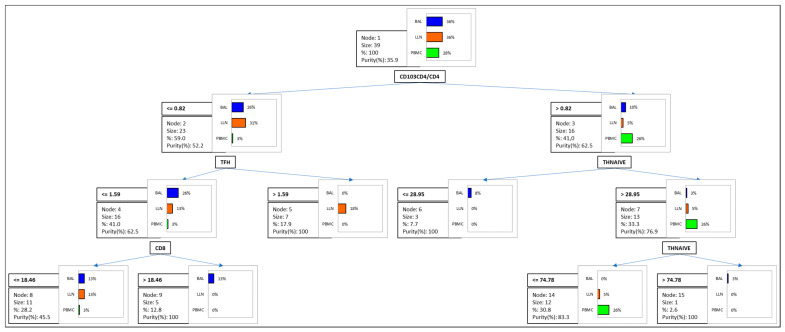
The diagram allows us to visualise the successive steps during which the regression decision tree algorithm identifies the variables to best split the three categories (peripheral blood mononuclear cells (PBMC), bronchoalveolar lavage (BAL) and lymph nodes (LLN) samples) of the dependent variables (lymphocyte subsets).

To determine the best clustering variables to distinguish PBMC, BAL and LLN samples, a decision-tree model (with cross-validation by confusion matrix) was used (Figure 4). The model showed CD4^+^ CD103^+^/CD4^+^ ≤0.82 and Tfh ≤1.59 for 41.0% of BAL samples, as well as CD4^+^ CD103^+^/CD4^+^ >0.82 and ThNaive >28.95 and ≤74.78 for 30.8% of PBMC samples.

## 4. Discussion

The present study reports an analysis of CD103 expression on follicular and regulatory T cell subsets in peripheral blood, BAL fluid and lymph node tissue from sarcoidosis patients. T-cell lymphopenia in peripheral blood typically observed in patients with sarcoidosis seems to be the result of enhanced recruitment into sarcoid lesions in nonlymphoid tissues [18]. A feature of sarcoidosis is the accumulation of CD4^+^ T lymphocytes in the granulomas [19]. As expected, CD4^+^ T cell percentages were lower in peripheral blood than in lymph nodes [12]. The role of CD4^+^ T cells in the immunopathogenesis of sarcoidosis is well established [19,20], whereas less is known about cytotoxic CD8^+^ T cells.

The multivariate analysis found that the variables which most influenced the peripheral blood compartment were high CD8^+^ and low ThReg, CD8^+^CD103^+^ and Tfh cell percentages. Our previous results concerning expression of CD103 in BAL from ILD patients depicted sarcoidosis as a disease in which this integrin is very much involved [6]. The logistic regression showed the variables that most influenced the three differential compartments were CD8, ThReg, CD8^+^CD103^+^ and Tfh. Our decision tree analysis demonstrated that the best clustering variables were the CD103^+^CD4^+^/CD4^+^ ratio and ThNaive percentages in peripheral blood, as well as the CD103^+^CD4^+^/CD4^+^ ratio and Tfh percentages in the alveolar compartment, sustaining the potential role of these immunological findings in the characterisation of sarcoidosis patients. 

A BAL cell pattern with a CD4/CD8 ratio > 3.5 has been postulated as a potential diagnostic indication of sarcoidosis [19] but has not been observed in LLN samples obtained by EBUS-TBNA [21]. Although our study confirmed this in our sarcoidosis population, a higher CD4/CD8 ratio was observed in lymph node tissue than in peripheral blood. Ruiz et al. found a higher CD4/CD8 ratio in BAL than in mediastinal lymph node tissue [22]. These findings were not comparable with ours because the authors did not phenotype their sarcoidosis patients as well as they did not perform the comparison analysis including peripheral blood samples [23].

Lung cells express many combinations and permutations of integrin heterodimers and it is now appreciated that integrins play important roles in many other biological processes [8]. Integrins could also mediate lymphocyte homing and migration into tissues [9,24]. Thus, there is still great interest in cellular biomarkers that could be related to inflammation and immune cell migration [12,25,26]. 

Among the integrins implicated in respiratory disorders, CD103 plays an important role in the selective retention of lymphocytes in mucosal tissues of the lung during disease progression [27]. The integrin CD103 is only expressed on lymphocytes of the intraepithelial phenotype [28].

In our sarcoidosis patients, double positive CD4^+^CD103^+^ cells were more elevated in peripheral blood than in the alveolar compartment, suggesting that the lower expression of CD103 on CD4 BAL lymphocytes may be related to their peripheral origin [29,30]. Various studies have suggested that a high CD4^+^/CD8^+^ ratio combined with a low CD103^+^CD4^+^/CD4^+^ ratio could be a promising BAL diagnostic marker of sarcoidosis [14,29]. This was confirmed by our results. The present results are the first to demonstrate time higher CD8^+^CD103^+^ percentages in the alveolar compartment than in peripheral blood and lymph nodes. Farber et al. demonstrated the existence of a small fraction of CD103^+^CD8 T cells in the lung [31].

The CD127 antigen is a 75–80 kDa type I transmembrane glycoprotein also known as IL-7 receptor α chain. It is a useful marker to identify memory and effector T cells. Its expression is downmodulated on Treg cells. In sarcoidosis patients, Th-reg (CD4^+^CD25^+^CD127^−^) cells are impaired in their ability to suppress granuloma development. According to our results, Th-reg cells accumulate in the alveolar compartment more than in peripheral blood, probably due to peripheral recruitment [32,33,34]. Although we did not find statistically significant differences in the distribution of Tc-reg, our study is the first to describe their increment in the alveolar compartment.

Conversely, Th effector (CD4^+^CD25^+^127^+^) and Th naive (CD4^+^CD25-CD127^+^) cell percentages were lower in the alveolar compartment than in peripheral blood and lymph node tissue because of the resistance of pathogenic effector T cells to suppression by Treg cells [35]. Assessment of CD127 expression on CD8-positive cells showed that Tc naive and Tc effector had a similar distribution to the abovementioned cell subsets.

We also evaluated CD103 expression on Th-reg cells: double-positive cell percentages were higher in peripheral blood than in BAL fluid and lymph nodes, suggesting a new paradigm for sarcoidosis. Interestingly, Tc-reg cells expressing CD103 were only detected in BAL samples from our sarcoidosis patients.

Tfh are a specialised subset of CD4^+^ T cells involved in the humoral adaptive immune response. They express the chemokine receptor CXCR5, which is necessary for their migration to B cell follicles rich in CXCL13 (B-cell chemoattractant) [36]. Ly et al. reported infiltration of Tfh cells into skin lesions of sarcoidosis patients, suggesting that circulating Tfh cells are recruited to affected tissues and localise in the periphery of new and early granulomas, where they play their inflammatory role in sarcoidosis [37]. Tfh cells are located in secondary lymphoid organs, including the tonsils, spleen and lymph nodes. These organs contain many lymphocytes separated into well-defined T- and B-cell zones. Our study is the first to reveal the enhancement of Tfh and B cells in LLN samples rather than in BAL and peripheral blood from sarcoidosis patients.

To our knowledge, the present study is also the first to describe Tfc cells expressing CD103 in ILD patients, including sarcoidosis. It has been reported that Tfc cells develop in response to viral infection and cancer, and can migrate to B cell follicles and eradicate virus-infected Tfh cells and B cells [38]. Notably, Tfc cells can also differentiate into T helper cells, which directly help B cells to form germinal centres and produce antibodies. Our sarcoidosis patients showed higher Tfc percentages in lymph nodes than peripheral blood and they were not detected in BAL samples. However, the alveolar compartment showed high Tfc-CD103 cell percentages compared to lymph nodes and peripheral blood, probably due to the chief ligand for CD103, E-cadherin, an adhesion molecule important for T cell homing to interstitial sites. E-cadherin expression in macrophages dampens their inflammatory responsiveness in vitro, but does not modulate M2-regulated pathologies in vivo [39]. Several studies of sarcoidosis have suggested that the triggering factor for inflammation is contact of a foreign antigen with antigen-presenting cells, leading to activation of T and B lymphocytes, which migrate to the inflammatory foci. If the antigen persists, macrophages undergo epithelioid differentiation and lymphocytes continue to migrate to the foci, forming granulomas [40]. 

Although our results contribute to understanding of T- and B-cell subsets in the pathogenesis and diagnosis of sarcoidosis, our study has some limitations, including the fact that definite cut-off values were not established because healthy donors could not be included for ethical reasons. Our findings warrant investigation in a multicentre study with a larger cohort.

## 5. Conclusions

Alterations in T- and B-cell immune responses have been reported in several studies of sarcoidosis, and one of its hallmarks is known to be the influx of T cells into the active sites of this disease. Our study is the first to report a link between the imbalance in circulating, alveolar and lymph node CD8^+^ and CD8^+^CD103^+^ T cells, ThReg, Tfh and ThNaive and the CD103^+^CD4^+^/CD4^+^ T cell ratio in the development of sarcoidosis. These findings shine a spotlight on the pathogenesis of sarcoidosis and may offer new predictors for diagnosis. Our study provides additional understanding for a personalised, and hopefully more effective treatment of sarcoidosis.

## Figures and Tables

**Table 1:** CD4, CD8, regulatory and follicular T cell subsets in bronchoalveolar lavage (BAL), peripheral blood mononuclear cells (PBMC) and lymph nodes (LLN) from sarcoidosis patients.

Parameters Mean ± SD	LLN	BAL	PBMC
CD4 (Th)	67.1 ± 25.5	66.3 ± 16.3	55.1 ± 15.4
CD4^+^CD103^+^	36.4 ± 22.6	37.0 ± 20.2	64.5 ± 25.1
CD4^+^CXCR5^+^ (Tfh)	0.755 ± 0.853	0.681 ± 0.724	0.302 ± 0.360
Tfh CD103^+^	3.59 ± 3.77	5.80 ± 6.00	3.27 ± 2.29
CD4^+^CD127^+^CD25^+^ (Th Effector)	3.26 ± 3.41	1.08 ± 1.02	3.67 ± 3.44
CD4^+^CD127^+^CD25^−^ (Th Naive)	43.8 ± 21.3	23.2 ± 21.1	51.5 ± 21.7
CD4^+^CD25^+^CD127^−^ (Th Reg)	7.01 ± 3.97	7.01 ± 5.15	4.78 ± 2.92
ThReg CD103^+^	34.2 ± 31.0	29.4 ± 28.8	60.8 ± 21.5
CD8 (Tc)	14.4 ± 12.6	21.8 ± 15.6	30.7 ± 12.8
CD8^+^CD103^+^	0.751 ± 0.964	0.183 ± 0.449	0.910 ± 1.11
CD8^+^CXCR5^+^ (Tfc)	0.0817 ± 0.200	0.0390 ± 0.123	0.216 ± 0.285
Tfc CD103^+^	0.0357 ± 0.0945	0.823 ± 1.15	0.134 ± 0.228
CD8^+^CD127^+^CD25^+^ (Tc Effector)	4.44 ± 4.39	1.32 ± 3.25	0.747 ± 0.865
CD8^+^CD127^+^CD25^−^ (Tc Naive)	21.4 ± 12.9	10.4 ± 10.8	16.7 ± 11.4
CD8^+^CD25^+^CD127^−^ (Tc Reg)	0.503 ± 0.649	1.01 ± 1.38	0.480 ± 0.565
TcReg CD103^+^	5.00 ± 11.2	47.7 ± 43.5	6.03 ± 13.6
CD4/CD8 ratio	4.85 ± 4.33	3.10 ± 3.60	1.80 ± 1.42

## Data Availability

The data presented in this study are available on request from the corresponding author.
